# Inflammatory bowel disease following anti-interleukin-1-treatment in systemic juvenile idiopathic arthritis

**DOI:** 10.1186/s12969-017-0147-3

**Published:** 2017-03-14

**Authors:** Boris Hügle, Fabian Speth, Johannes-Peter Haas

**Affiliations:** German Center for Pediatric and Adolescent Rheumatology (GCPAR), Gehfeldstrasse 24, 82467 Garmisch-Partenkirchen, Germany

**Keywords:** Systemic juvenile idiopathic arthritis, Inflammatory bowel disease, IL-1 Antagonists

## Abstract

**Background:**

Inflammatory bowel disease can develop in the context of some rheumatic diseases in childhood, including juvenile idiopathic arthritis (JIA). Inflammatory bowel disease (IBD) is frequently associated with other immune-mediated diseases; however, systemic onset JIA (sJIA) has not previously been connected to IBD. Treatment of sJIA has significantly changed in recent years, possibly causing changes in inflammatory patterns. Therefore, data from the German Center for Pediatric and Adolescent Rheumtology from 2010 until 2015 were analyzed by retrospective chart review.

**Findings:**

Eighty-two patients with confirmed diagosis of sJIA were found. Of these, three were identified with a diagnosis of IBD confirmed by colonoscopy (two cases of Crohn’s disease, one case of ulcerative colitis) 0.8 – 4.3 years after diagnosis. All three were treated with IL-1 antagonists (anakinra in two cases, canakinumab in one case) and were well controlled for sJIA symptoms at time of diagnosis of IBD

**Conclusions:**

IBD seems to be a rare, but possible complication of sJIA. Treatment with IL-1 antagonists might be a relevant factor for a switch in the clinical phenotype of the underlying inflammatory process.

**Electronic supplementary material:**

The online version of this article (doi:10.1186/s12969-017-0147-3) contains supplementary material, which is available to authorized users.

## Findings

Systemic-onset juvenile idiopathic arthritis (sJIA) is an autoinflammatory disease characterized by increased levels of interleukin(IL)-1 and IL-6 [1]. Anakinra and canakinumab are both medications used successfully in sJIA, aiming to neutralize the effects of IL-1 [2, 3].

Inflammatory bowel diseases (IBD) are associated with several immune-mediated diseases, with almost a quarter of patients showing first symptoms during childhood or adolescence [4]. While unspecific mild bowel inflammation has been described in sJIA, IBD has not been observed as an extrarticular manifestation [5, 6].

We present three pediatric cases with confirmed diagnosis of sJIA who developed IBD diagnosed by histology and coloscopy during treatment with IL-1 antagonists.

## Patients and Methods

The database of the German Center for Pediatric and Adolescent Rheumatology was searched for all sJIA patients (*n* = 82) with a confirmed diagnosis of IBD from 2010 until 2015. Four patients were identified; one was excluded as histology showed only unspecific round-cell infiltrate. Chart survey was used to extract demographic data, date of diagnosis of sJIA and IBD, clinical and laboratory data and medications.

## Case descriptions

Clinical and laboratory data of the cases at time of diagnosis of sJIA and of IBD are described in Tables 1 and 2, respectively. (Additional file 1: Table S1 and Additional file 2: Table S2).

**Table 1:** Demographics of the patients, clinical and laboratory data at time of diagnosis of sJIA, and time and dose of anti-IL-1 medication

Patient	1	2	3
Gender	m	m	f
ANA/RF/HLA-B27	neg/neg/neg	neg/neg/n.d.	neg/neg/neg
Age at diagnosis	14.8	15.8	6.8
ILAR criteria for subtype	Fever, typical rash, splenomegaly, pericardial effusion	Fever, rash, pericardial/pleural effusion, hepatomegaly	Fever, rash, pleural effusion, hepatomegaly splenomegaly
Affected joints	Polyarthritis	Polyarthritis	Elbows, hips, wrists
Leukocyte count	9100/mm^3^ (4800–10000)	9100/mm^3^ (4500–12500)	16800/mm^3^ (4800–10000)
Platelet count	294000/mm^3^ (150000–450000)	208000/mm^3^ (154000–386000)	575000/mm^3^ (150000–450000)
Ferritin	2926 μg/l (30–400)	32722 ng/ml (22–322)	297–1197 μg/l (30–400)
C-reactive protein	7.4 mg/dl (0.2–1.0)	28.54 mg/dl (<0.3)	3.36 mg/dl (0.2–1.0)
Bone marrow biopsy findings	Negative	MAS	-
Start of IL1-treatment, years after diagnosis	0 years	2.2 years	0.3 years
IL-1 agents, initial dose	Anakinra 1.3 mg/kg	Canakinumab 1.8 mg/kg	Anakinra 1.1 mg/kg

normal values in parentheses

*neg* negative, *n.d.* not done

**Table 2:** Colonoscopy findings, clinical and laboratory data at time of diagnosis of IBD of the patients

Patient	1	2	3
Time between diagnoses of sJIA and IBD	0.9 years	4.4 years	4.7 years
Time between start of Anti-IL1 agent and IBD	0.9 years	2.2 years	4.4 years
Anti-IL1 agents, dosage	Anakinra, 1.3 mg/kg	Canakinumab, 1.8 mg/kg	Anakinra, 0.7 mg/kg
Other medications	None	Cyclosporin A	None
Colonoscopy, macroscopic findings	Active, ulcerous ileitis and colitis, consistent with Crohn’s disease	Active colitis from sigmoid to coecum, consistent with Crohn’s disease	Colitis in colon descendens, consistent with ulcerative colitis
Colonoscopy, microscopic findings	Granulomatous inflammation	Inflammatory infiltrate	Inflammatory infiltrate
Diarrhea	Present	Present	Present
Macroscopic blood in stool	Absent	Absent	Present
Ferritin	138 ng/ml (9–80)	154 ng/ml (30–400)	10 ng/ml (30–400)
CRP	12.1 mg/dl (< 0.8)	4.67 mg/dl (0.02–1.0)	0.44 mg/dl (0.2–1.0)
Calprotectin/stool	Not available	877 μg/g (<50)	1264 μg/g (<50)

normal values in parentheses

*Case I* is a Caucasian male. He presented at age 14 years and 10 months with high quotidian fever with associated diffuse exanthema, splenomegaly, mild pericardial effusion and polyarthritis. Infectious workup showed elevated IgG and IgM antibodies against Parvovirus B19. He did not complain about abdominal symptoms and had a normal bowel ultrasound except for splenomegaly. He received methylprednisolone pulse therapy and indomethacin and was started on anakinra, with rapid remission within 3 months.

After 11 months on anakinra he developed increasing abdominal pain and diarrhea. Stool cultures were negative. Colonoscopy showed ileitis and discontinuous inflammation in the ascending colon. Histology confirmed granulomatous inflammation. With a diagnosis of Crohn’s disease he was started on azathioprine and prednisolone, while anakinra was tapered. He presented 3 months later with fevers and markedly elevated inflammatory parameters without diarrhea. After excluding systemic inflammation or infection, his colonoscopy was repeated and returned a result of highly active Crohn’s disease. Treatment with infliximab and methotrexate was started, leading to rapid and ongoing remission 8 months later.

*Case II* is a Caucasian male, who presented at age 15 years and 10 months with fevers, arthritis and exanthema and developed polyarthritis, pericardial and pleural effusion within a few weeks. At that time, he had borderline positive IgM antibodies against Coxsackie virus, Adenovirus and Epstein-Barr virus. He was treated with NSAID and steroids, but developed two episodes of macrophage activation syndrome, with recurrence on cyclosporin after the first episode. Three months prior to the second episode, he was diagnosed with Influenza A. Twenty-three months after diagnosis he was started on anakinra after methylprednisolone pulses which led to brief improvement. Three months later he presented with severe signs of systemic arthritis and elevated inflammatory parameters. Treatment with tocilizumab showed no improvement, but canakinumab – in addition to cyclosporine and low dose steroids – led to rapid and lasting remission over the next 2 years.

After 24 months on canakinumab he presented with fatigue, arthralgias without arthritis and elevated C-reactive protein, with diarrhea and weight loss. Increasing doses of canakinumab and prednisolone did not lead to improvement. Infectious work-up showed again borderline positive IgM antibodies against Coxsackie, but negative stool cultures. Colonoscopy showed active colitis extending from proximal sigmoid to the cecum with several, partly confluent ulcerations. The iliocecal valve was gaping, and the terminal ileum exhibited erythema and punch defects of the mucosal membrane. Duodenal biopsy showed edematous and coarse villi, with detachment of epithelium and mostly neutrophilic inflammatory infiltrate. Even without granuloma formation, this was interpreted as Crohn’s disease, supported by positive antibodies against *Saccharomyces cerevisiae* (ASCA). Canakinumab was discontinued, and he was started on budenoside and infliximab, which led to rapid remission, ongoing 18 months after diagnosis of IBD.

*Case III* is a Caucasian male of mixed German and Turkish origin. He presented at age 6 years and 10 months with fevers, arthritis, exanthema, pleural effusion and oligoarthritis. An extensive infectious workup showed no result. He was treated with indomethacin, steroids and methotrexate. He presented 4 months later with arthritis, fevers and elevated inflammatory parameters. Treatment with steroids and etanercept showed no effect after 3 doses, so he was switched to anakinra, leading to prompt improvement of clinical signs and laboratory parameters.

He entered long lasting remission, but started to develop mild abdominal symptoms after 3 years on anakinra and methotrexate. Methotrexate was discontinued 6 months later, and in the following months he showed increasing abdominal pain and blood in his stools. Stool cultures were negative. He received a colonoscopy 4 years after starting anakinra, which showed severe inflammation of the colon descendens consistent with ulcerative colitis, with histology showing edema of the lamina propria with inflammatory lymphocytic and neutrophilic infiltrate, and also focal inflammation in the crypt walls. He was continued on low dose anakinra (0.5 mg/kg) and started on mesalazin and azathioprine, with ongoing mild gastrointestinal complaints on last follow-up.

## Discussion

Here we present three patients with confirmed diagnosis of sJIA who developed signs of IBD on treatment with anti-IL1 agents, either anakinra or canakinumab. All cases fulfilled ILAR criteria for sJIA at the time of diagnosis, and none had any clinical signs of IBD at that time. Although the age range in these patients falls well within the described spectrum, the majority of patients with sJIA initially present below the age of 5 years. All patients developed typical manifestations of Crohn’s disease or ulcerative colitis, where monoclonal TNFα antibodies or DMARDS were required to gain disease control (for comparison, see Table 3). The one patient receiving low-dose anakinra in fact continued to have low grade abdominal symptoms, possibly indicating an ongoing pro-inflammatory effect of anti-IL1 medication.

**Table 3:** Similarities to and differences between the presentation of systemic juvenile idiopathic arthritis and inflammatory bowel disease in children

	Juvenile Idiopathic Arthritis	Inflammatory Bowel Disease
Onset of the disease	Acute onset with high, quotidian fevers,	Typically subacute illness with fatigue, anemia, and weight loss. Occasionally more fulminant presentation.
Gastrointestinal symptoms	Frequently abdominal pain, also nausea and anorexia	Loose stools and/or bloody diarrhea, abdominal pain, tenesmus
Muskuloskeletal symptoms	Initially, mild oligoarticular arthritis, frequently severe polyarthritis over the course of the disease	Nonerosive, asymmetric arthritis, affecting the large joints, parallel to intestinal involvement
Skin manifestations	Initial presentation with evanescent, salmon-colored, cutaneous eruption	Erythema nodosum, pyoderma gangrenosum
Laboratory abnormalities	Anemia, reactive thrombocytosis, markedly elevated erythrocyte sedimentation rate and C-reactive protein. Significantly elevated ferritin. Typically negative antinuclear antibodies and rheumatoid factor.	Anemia, elevated erythrocyte sedimentation rate and C-reactive protein, depressed albumin level, occult blood in the stool, elevated fecal calprotectin. Ferritin typically low or normal. Typically negative antinuclear antibodies and rheumatoid factor.

Few reports exist of patients with sJIA developing IBD. Twenty-nine patients with various subtypes of JIA, including three with sJIA, have been linked to IBD on treatment with etanercept, and one on infliximab [7–9]. All three patients developed Crohn’s disease between 5 and 12 years after diagnosis of sJIA on treatment with etanercept; all patients went into clinical remission after discontinuation of etanercept and treatment of IBD. A recent survey from the German biologics registry did find IBD in eleven of 3,071 patients with JIA, but none with sJIA [10]. Endoscopic studies on 27 JIA patients with gastrointestinal symptoms also found ulcerative colitis in five patients (19%), but none with sJIA [5]. Phenotypes of rheumatic diseases can evidently change over time, especially in childhood. sJIA changes from IL-1 driven acute febrile sJIA to IL-17 driven chronic active sJIA over time [1]. In endoscopic mucosal samples, IBD similarly exhibits a strong T_H_1 phenotype in early lesions and switches to a T_H_17 phenotype, with marked increase in IL-17A, and induction of IL-6 and IL-23; it can occur subsequent to different categories of JIA as well as spondyloarthropathies [11, 12]. IBD, especially Crohn’s disease, is considered an NF-κB activation disorder as part of the spectrum of autoinflammatory diseases, where cross-overs have frequently been observed – a change of sJIA into an IBD phenotype therefore seems at least possible in theory [13]. In fact, a recent study of mucosal biopsies in 33 JIA patients with gastrointestinal symptoms included eight with sJIA, with two patients with mild eosinophilic infiltration in the colon, and one 4 year old patient with active gastrointestinal inflammation [6].

All three patients were exposed to IL-1 antagonists when developing symptoms of IBD. IL-1 is known to be expressed at high levels in colonic biopsies from patients with IBD, supporting the hypothesis of a pro-inflammatory role. Animal models of colitis both in rabbits and rats improve with blockade of IL-1 [14]. However, recent mouse models show a differentiated role for IL-1α and IL-1β, where IL-1α, produced mainly by colonic epithelial cells, acts in an inflammatory fashion [15]. In comparison, IL-1β, produced mostly by myeloid cells, promotes healing and repair in colonic tissue; IL-1β deficient mice show more severe colitis with failure of repair mechanisms [16]. Anakinra blocks both IL-1α and IL-1β, but in the mouse model described, experimental specific IL-1α antibodies were significantly more efficacious in reducing severity of colitis compared to external IL-1 receptor antagonist (IL-1RA). Canakinumab by design specifically targets IL1-β, with no effect on IL-1α. Disturbance of the IL1-1α/β equilibrium by these medications might possibly create a pro-inflammatory environment in the colon, leading to IBD in selected cases. Patients with active IBD have in fact been shown to have increased levels of IL-1RA [17]. So far, no clinical trials have been reported for IL-1 antagonists in IBD, but one case of IBD worsening with anakinra treatment has been described [18]. One patient with sJIA in a randomized clinical trial for anakinra did also in fact develop Crohn’s disease [19].

Gastrointestinal symptoms in JIA are not uncommon, with several series describing results from gastrointestinal investigations [5, 6]. The majority of cases, including those with sJIA, show mild, nonspecific inflammation, frequently with a prominent eosinophilic infiltrate, with more severe inflammation in only a few cases. It is possible that the three cases presented here represent the extreme end of this spectrum. However, all three patients had ongoing, severe abdominal manifestations over several months while sJIA symptoms were well controlled. The three patients had extensive infectious work-up during diagnosis, which showed positive IgM antibodies for various pathogens, whose impact on the disease progression and change in phenotype is unclear.

This is a case series; therefore, any conclusions about an underlying pathological mechanism would be premature. The impact of medications on the development of IBD is unclear: the interval between IL-1 blocker treatment and IBD manifestations is highly variable, and it is possible that methotrexate withdrawal in case 3 lead to uncovering of a previously subclinical IBD. However, three cases of IBD in a single tertiary center cohort of 82 patients argues against mere coincidence.

In summary, this is the first report of sJIA patients developing IBD in temporal connection with IL-1 antagonist treatment. It is not clear if these cases are coincident, represent a change in patterns of inflammation of sJIA or are a consequence of treatment. Further efforts should aim at collecting more patients and elucidating the underlying disease processes.

## Additional files

Additional file 1: Table S1. Supplementary clinical and laboratory data for the patients at time of diagnosis of sJIA. (DOCX 23 kb)
Additional file 2: Table S2. Coloscopy results of the 3 patients, translated and summarized from the German original. (DOCX 23 kb)
