# Supplementary material for: Inflammatory bowel disease following anti-interleukin-1-treatment in systemic juvenile idiopathic arthritis
Source: Pediatr Rheumatol Online J. 2017 Mar 14;15:16. doi: 10.1186/s12969-017-0147-3 (PMC5348783; doi:10.1186/s12969-017-0147-3)
Supplement: Additional file 1: Table S1. — Supplementary clinical and laboratory data for the patients at time of diagnosis of sJIA. (DOCX 23 kb) [file 12969_2017_147_MOESM1_ESM.docx]

**Additional file 1: Table S1** Supplementary clinical and laboratory data for the patients at time of diagnosis of sJIA

| Patient | 1 | 2 | 3 |
| --- | --- | --- | --- |
| Fever, duration* | 37 days | 15 days | 36 days |
| Fever, spikes | twice daily | not described | daily |
| Temperature max/min | 40.0°C/35.6°C | 39.0°C/36.8°C | 39,6°C/36.5°C |
| Pleuritis, mode of diagnosis | n/a | ultrasound | ultrasound |
| Pericarditis, mode of diagnosis | echocardiography | echocardiography | echocardiography |
| Hepato-/Splenomegaly, mode of diagnosis | ultrasound | ultrasound | ultrasound |
| Exanthema, description | small macules, whole body,  evanescent, pruritus | small macules, trunk and extremities | small macules, mostly trunk |
| Arthritis, duration* | 5 weeks | 3 weeks | 5 weeks |
| Lymphadenopathy | cervical | cervical and submandibular | cervical |
| Albumin at diagnosis | 3.63 g/dl | not available | 3.36 mg/dl |
| ESR at diagnosis | 49 mm/hr | 47 mm/hr | 83 mm/hr |

*prior to antiinflammatory therapy (corticosteroids or anti-IL1 agents)
