# Supplementary material for: Inflammatory bowel disease following anti-interleukin-1-treatment in systemic juvenile idiopathic arthritis
Source: Pediatr Rheumatol Online J. 2017 Mar 14;15:16. doi: 10.1186/s12969-017-0147-3 (PMC5348783; doi:10.1186/s12969-017-0147-3)
Supplement: Additional file 2: Table S2. — Coloscopy results of the 3 patients, translated and summarized from the German original. (DOCX 23 kb) [file 12969_2017_147_MOESM2_ESM.docx]

**Additional file 2: Table S2** Coloscopy results of the 3 patients, translated and summarized from the German original

| Patient | Findings |  |  |  |
| --- | --- | --- | --- | --- |
| Case I | Macroscopic results: Typical endoscopic findings of Crohn’s disease from transverse colon up until the terminal ileum.  Histology: florid ulcerative ileitis and colitis in the terminal ileum, cecum and ascending colon with decreasing inflammatory activity reaching from the transverse colon until the sigmoid. With moderately disturbed crypt achitecture, a small granuloma in the terminal ileium and discontinuous expansion of the inflammation, this can be interpreted as Crohn’s disease. | | | |
| Case II | Macroscopic results: Active colitis in an area extending from proximal sigmoid to cecum with several, partly confluent ulcerations. Gaping iliocecal valve; the terminal ileum shows erythema and punch defects of the mucosal membrane.  Histology: Duodenal biopsy with edematous and coarse villi, aggregation of lymphoid cells and follicles. In some locations detachment of epithelium, here inflammatory infiltrate, mostly of neutrophils, is found.  Colon biopsy with spotty inflammatory infiltrate, mostly neutrophils, with leukocyte extravasation and epithelial detachment. No granuloma formation.  Interpretation is a moderately active, partly erosive ileitis and colitis, most active in the ascending colon, consistent with Crohn’s disease, but without unequivocal granuloma formation. | | | |
| Case III | Macroscopic results: erythematous mucous membrane in rectum and distal sigmoid, without visible vasculature, with erythema and bleeding on contact, several fibrinous spots, consistent with ulcerative colitis.  Histology: In 3 of 7 biopsy samples mild to moderate colitis with edema of the lamina propria, follicular lymphocytic infiltrate and focal infiltation of neutrophils, also found in the crypt walls. | | | |
